# Book Review

**Published:** 1925

**Authors:** 


### Reviews of Books.

**Orations and Addresses.** By Sir John Bland-Sutton. Pp. 160. London: William Heinemann. 1924. 57 Illustrations. Price 10s. 6d. net.—We have read with great pleasure and interest this volume, comprising a dozen addresses given on various subjects by this distinguished writer—from that on Cranial Morphology (*Journal of Anatomy and Physiology*, 1887–88, xii. 28) to the Habits (Ecology) of Tumours, delivered before the Cambridge Medical Society in 1923. The Hunterian Oration and the chapters on Choroid Plexuses and Psammomas, Pacchionian Tumours and Cranial Exostoses, as well as the description of the War Pathological Collection of the Royal College of Surgeons are particularly noteworthy. The researches of W. G. Penfield on the origin of cranial exostoses (1921) are included: Pacchionian villi erode the dura mater and cranium and cause a complete rearrangement of the osseous structure. The bone-forming elements lay down bone in the substance of the neoplasm. The pathological originality of the author, his versatility and lucidity, are apparent on every page; the woodcuts are numerous and there is an ample index.

**Leprosy.** By Sir Leonard Rogers, C.I.E., M.D., F.R.C.P., F.R.C.S., F.R.S., I.M.S. (retired) and Ernest Muir, M.D.,

F.R.C.S. Edin. Pp. xii., 301. Bristol: J. Wright & Sons Ltd. 1925. Price 12s. 6d.—For many years the problems of this fell disease have puzzled medical science and a large part of them yet remain unsolved. Fifty years ago the discovery of the bacillus lepræ by Hansen was a great step forward. This led to great alteration in ideas with regard to the epidemiology and etiology of the complaint, and the old theory of heredity had to give place to the now accepted one of infection. However, knowledge has not been advanced as much as was expected ; it has proved impossible to grow the bacillus lepræ outside the body, and although some workers have claimed to have done so, closer investigation has shown that they were not working with a true strain or some other fallacy has been found in their work. The period of incubation is still doubtful, for few if any cases on record allow of a definite period being affixed, the length of time from possible infection to first manifestation being variously reported from six months to ten years, the length of the period being apparently affected by many factors such as climate, conditions the patient is living under, his state of health, if subject to other diseases, etc., etc. Again, the nature of the primary manifestations is not clearly established ; they generally escape the notice of patients, who frequently do not come under notice until the disease is well established. In the treatment of the disease many and various drugs and methods have been employed with little or no curative effect, until late years, when modern methods, in which the authors of this book have played a large part, have proved "effective in clearing up the symptoms and infectivity of most early and some more advanced cases." In view of this advance in treatment the authors consider that a review of our knowledge and the facts in our possession concerning leprosy and a reconsideration of the whole case is necessary. For this purpose in their book they summarise the most important previous literature having a practical bearing on the subject, and give a clinical account of leprosy based on a large personal experience. The history of the disease is full, and gives the story of its spread and subsequent decline in Western Europe and also tells of its spread to the Western Hemisphere. The distribution, communicability and etiology are duly and fully dealt with. Prophylaxis receives its due meed of attention and many practical points are discussed, their value demonstrated and made clear. This part of the book should prove of considerable value to those in administrative positions. The clinical description of the disease is based on the authors' own large personal experience and is therefore the more valuable. They divide the types of disease into three classes, viz. skin, nerve and mixed, considering that confusion has been caused by the usual classification of nodular or tubercular and maculo-anæsthetic types. They divide the individual lesions in a similar way, defining a skin lesion as a skin area in which lepræ bacilli can be found on bacteriological examination, but in which there is no anæsthesia to light touch; and a nerve lesion as one indicated by an area of skin in which bacilli cannot be found on bacteriological examination but in which there is anæsthesia to light touch. All their descriptions are based on this "clear-cut description." The clinical picture is clearly depicted, and the points emphasised in the letterpress are made more clear by good illustrations taken from the authors' own cases. Diagnosis, based on the points dealt with in the clinical description, is given full consideration and should help in the early diagnosis of cases, a point so important when dealing with this disease, which is duly accentuated in the book. A historical review of past treatment is given, and this leads up to the present-day one used and advocated by the authors—esters of chaulmoogra, hydnocarpus and other oils. The procedure is fully explained, the method of the preparation of the esters, the method of their injection and dosage, the effects on the patient, reaction, etc., etc., are all in turn discussed. The selection of suitable cases also is fully dealt with and the views of the authors made clear. As well as special treatment the importance of general treatment is impressed on the reader, and many points emphasised, such as the necessity of the removal of the exciting and predisposing causes. The book is a most readable one, full of good matter; the personal element so marked throughout, especially in the sections on prophylaxis and treatment, makes it all the more valuable and enjoyable. In conclusion, we are sure the desire expressed by the authors that it may prove of service to workers in affected countries will be fully realised.

**The Bacteriology of Food.** By Clement Dukes, M.D., M.Sc., D.P.H. Pp. vii., 180. London: H. K. Lewis & Co. 1925. Price 7s. 6d. net.—Provides a fairly complete account of the bacteriology of most of the common foods and of water. In addition to a brief discussion on food preservation includes water, milk, the various condensed and dried milks, butter, cheese, canned foods, alcoholic beverages, bread, vinegar and an account of bacterial food poisoning. While written primarily to meet the needs of science students studying bacteriology as one of the final subjects in the B.Sc. degree of London University, it will be found of general interest to medical men and students. Although not a laboratory text-book, it covers fairly well the laboratory methods in vogue for the examination of the foods indicated. An account of the bacteriology of shellfish might well have been included. The largest section deals with milk, and supplies a clear account of this subject, which is well up to date. Some omissions may be mentioned. Bovine mastitis as a source of infection in human sore throat outbreaks is mentioned, but its importance is underrated, while no mention is made of the fact that in a number of instances Gærtner group bacilli have been isolated from the milk of individual cows which have been responsible for food poisoning outbreaks. *B. melitensis* in relation to its spread by milk is not mentioned nor is its relationship to *B. abortus*. The account of the different types of tubercle bacilli is very short. It is difficult, however, to include even in forty-six pages a comprehensive account of the bacteriology of such a very complex fluid, and on the whole the author is to be congratulated upon his success. A welcome section deals with the bacteriology of canned foods, as these have hitherto been for the most part neglected in general text-books upon food or only treated from the chemical aspect. In general the author has succeeded well in his object, and readers will find an attractively written book giving a great deal of useful information. Some of it, such as the parts played by bacteria in the production of butter, cheese, vinegar, etc., is not readily available in English text-books. Several pages are also devoted to the subject of fermented milk, the bacterial nature of the effervescent types, such as *Kefir* and *Koumiss*, as well as the non-effervescent type as exemplified by *Yogurt* being explained. The author characterises the soured and fermented milk treatments as a craze, and does not advance any bacteriological data favouring the practice as of real therapeutic utility.

**An Introduction to Dermatology.** By Sir Norman Walker. Eighth Edition. With 92 Plates and 80 Illustrations in the text. Pp. xviii., 373. Edinburgh: W. Green & Son Ltd. 1925. Price 20s. net.—For many years we have recommended Norman Walker's *Introduction to Dermatology* as the best handbook for students and general practitioners. The eighth edition has been largely re-written, and by the omission of some of the rarer skin diseases more adequate accounts can be given of the common conditions. The illustrations are still being added to, and their excellence seems to increase with each edition. The term "Autophytica" for "self-inflicted" has nothing to commend it; and we disagree entirely with the author's excuse for the Scots word "Kenspeckle" that there is no English word for it. We suggest that in the next edition the word "conspicuous" might be employed as the earlier form of the Lowland Scots corruption. These are the only faults to be found with a book of outstanding merit.

**Chronic Intestinal Stasis.** By ALFRED C. JORDAN. Pp. xi., 230. London : Henry Frowde, Hodder and Stoughton. 1923. Price 25s.—There is a tremendous amount of work and information in this book. The author is to be congratulated on his investigations of intestinal conditions, and though all will not agree with his deductions, it is a book of the utmost value.

**A Manual of Practical X-ray Work.** By JOHN MUIR. B.Sc., M.B., Ch.B., B.Sc. Pp. x., 524. London: William Heinemann. 1924. Price 31s. 6d.—A very excellent treatise on X-rays in general. The latter half of the book, dealing with diagnosis and reading of X-ray films, is particularly lucid and helpful. Should be carefully read by those interested in this branch of medicine.

**Epidemic Encephalitis.** By ARTHUR J. HALL, M.A., M.D., F.R.C.P. Pp. xii., 229. Bristol : John Wright & Sons Ltd. 1924. Price 12s.—This is a comprehensive study of our present knowledge of Encephalitis, a disease which is proving itself a serious problem to all students both of medicine and sociology. Although not a new disease, it has never within recent times shown such a wide incidence or such serious effects. In view of the latter factor the recognition of the frequent occurrence of slight cases and the precautions against carriers of naso-pharyngeal infection are all important. The low grade but definite contagiousness of the disease must be remembered not only at the time of the original attack, but also during the progress of the residua and recrudescences. A full description of the pathology and characteristics of the virus is given and the symptomatology is adequately dealt with. In the discussion of residua the mental changes are given prominence, but in view of their tremendous social import it is doubtful if the matter is even here sufficiently stressed. The short section on treatment only serves to indicate how barren are our resources in face of this menace. Finally there is a complete bibliography up to the date of publication of seventy-four pages, in addition to the references at the end of each chapter.

**Acute Infectious Diseases.** A Handbook for Practitioners and Students. By J. D. ROLLESTON, M.A., M.D. (Oxon.). Pp. vii., 376. London: William Heinemann (Medical Books) Ltd. 1925. Price 12s. 6d.—This is a very valuable addition to our text books, dealing with the commoner infectious diseases. The writer's wide experience has made it possible for him to speak with authority upon many contentious points, and every chapter is full of interest and information. The pages devoted to treatment will be especially appreciated by practitioners, for the directions given are always clear, brief, and practical. Space is also devoted to a description of some of the less common infectious diseases—Paratyphoid Fever, Vincent's Angina, the Fourth Disease, and Erythema Infectiosum. It is interesting to note that the author regards cases of Fourth Disease as probably examples of mild scarlet fever or rubella.

**A Contribution to the Study of Pernicious Anæmia and Aplastic Anæmia.** By ARTHUR SHEARD, M.D. Pp. vii., 94. Bristol: John Wright & Sons Ltd. 1924. Price 7s. net.—In this book—a reprinted M.D. thesis—Dr. Sheard offers a lucid account of modern views on pernicious anæmia. He conforms to the belief in achlorhydria as an essential preliminary, the disease being actually excited by some infection generated within the gastro-intestinal tract. A case of aplastic anæmia is also described fully and reviewed. Dr. Sheard thinks the two diseases essentially different; the one is an excessive hæmolysis due to certain constant morbid processes, while the other is a failure of hæmopoiesis due to various causes. There are some interesting appendices, one of them summarising what is known as to the familial factor in prenicious anæmia. No one interested in diseases of the blood should neglect to read this little monograph.

**Tuberculosis: Its Prevention and Treatment.** By JOHN LAIRD, L.R.C.P. and S.I. Second Edition. Pp. 130. Bristol: John Wright & Sons Ltd. Price 5s. 6d.—Dr. Laird has written a book that has the merit of being both short and readable. His thesis is the curability of tuberculosis by simple home treatment. There is no practitioner, whether special or general, who will not gain something from making the acquaintance of these few pages.

**The Practical Medicine Series. Volume I.: General Medicine. Series 1924.** Chicago: The Year Book Publishers. Pp. 735. Price $3.—This is an American publication after the style of the *Medical Annual.* Though the American work occupies a relatively large fraction, that of other countries is included. The whole of medicine is reviewed, but in an unbalanced manner. There is little reference to nervous diseases, but the intention may be to treat this in another volume. There are many minor points which will annoy the reader, especially those of this country; the comments of the editors are in the vernacular, and though amusing and very much to the point, they appear out of place in a scientific work. Misprints are rather numerous. Paragraphs on the same subjects are not always consecutive, and repetitions are frequent at longer or shorter intervals. Very short notices or complete omissions of important foreign work are to be regretted; thus, Caronia's work on Scarlatina had more in its favour than that of the Americans' Dick, but it is accorded a very brief mention. Much work on the etiology of Addison's anæmia by Seyderhelm and others is not noted. In spite of these defects the book is of interest and value. The sections on diseases of the ductless glands and that on circulatory diseases are particularly good, not so much for the notices they contain, but for the amusing and sound comments appended by the reviewers; those of Preble in particular, while very American and hyper-caustic, are well worth reading. The book forms a moderately up-to-date review of medicine with a strong bias towards the American schools. It should be of help to the practitioner who desires to keep abreast of modern medical science, and for those who believe themselves not in need of such assistance it may still make an appeal as medical literature of much interest and yielding some amusement.

**Modern Operative Surgery.** Edited by H. W. CARSON, F.R.C.S., Senior Surgeon to the Prince of Wales' Hospital, Tottenham. In two volumes: Volume 1, with 365 Figures and two Plates, pp. 784; Volume 2, with 370 Figures and four Plates, pp. 784. London: Cassell & Co. Ltd. 1925. Price £3 3s.—This work, which is a successor and companion to Choyce's *System of Surgery*, is a most welcome addition to our English surgical literature. Its aim is to give a practical and authoritative account of all modern surgical operations. Each section is written by one who is an acknowledged authority on the subject. As is the case in all composite works, some sections are of much greater value than others. Anæsthesia is by Blomfield, and gives a short but practical account of the subject with due notice of spinal, regional and colonic anæsthesia. Sir Henry Gauvain contributes a most useful article on surgical tuberculosis, dealing chiefly with conservative methods. The chapters on general orthopædics and joints, by Elmslie and Verrall respectively, are astonishingly short, and both in matter of illustrations and text quite out of proportion to the rest of the book. Operations on tendons and amputations by Elmslie are good and practical, and in the latter section the omission of obsolete classical methods is a welcome improvement on other large systems. The sections on bone surgery (fractures, bone-grafting, etc.) is by Hey Groves, and gives an up-to-date account of modern operative methods. Thoracic operations by Warren and spinal cord surgery by Walton are on usually accepted lines and call for no special comment. The articles on nerve and vascular surgery by Platt and Drummond respectively are both very excellent presentations of the subjects, embodying the recent lessons of military surgery. Sampson Handley writes on the general surgery of malignant disease and that of the breast in particular. In the latter he regards the use of radium as necessary and he employs drainage very extensively. The chapters on abdominal operations have been written by Carson himself, and these represent the modern practice of English surgeons very faithfully, the works of Moynihan, Sherren and Miles having been freely drawn upon with due acknowledgment. The other most notable sections in the book are those dealing with the urinary organs by Sir John Thomson-Walker and plastic surgery by Gillies. Chapters on special regional surgery, the brain, ear, eye, nose, larynx, etc., are by well-known specialists. The book is very well illustrated, and uniformity of style is secured by all the figures having been specially drawn for this work by Mr. George Dupuy. Both editor and publishers are to be warmly congratulated on the production of a very notable achievement, namely an authoritative work of moderate size and expense, which nevertheless embraces the entire field of modern operative surgery. We confidently expect that in the next edition the orthopædic section will be more adequately dealt with.

**On the Breast.** By DUNCAN C. L. FITZWILLIAMS, C.M.G., M.D., Ch.M., F.R.C.S. Pp. xv., 440. London: William Heinemann. 1924. Price 30s.—To those in search of an up-to-date book on the diseases of the breast this may be cordially recommended. Much of the substance of the book of course follows stereotyped lines, clearly expressed and interestingly written and well illustrated. The principal value of this particular monograph, in competition with its rivals, lies in its excellent references to old and out-of-the-way cases, and in its capable criticism of Sampson Handley's permeation theory of the spread of cancer from the mammary gland. We are glad to see the use made of Sir Lenthal Cheatle's work on the "prœmial breast," and of Piney's researches on bone metastases. The advice given as to treatment of the various conditions described follows sound and well-travelled lines. The pathology, nevertheless, strikes us as being a little old-fashioned, in the light of the work of the best modern American pathologists such as Ewing. There are some very interesting problems, such as the rarity of cancer of the female breast in Japan, the evidence for the apparent special susceptibility of the mammary gland which has never functioned, and the remarkable influence exerted by X-rays on the encephaloid type of cancer, which are not referred to in the book, but which were recently discussed in this journal.

**Facial Surgery.** By H. P. Pickerill, C.B.E., M.D., M.S. Pp. xvi., 162. Edinburgh: E. S. Livingstone. 1924. Price 21s. net.—The surgeon who takes *Facial Surgery* for his guide in operations on the face will win deep gratitude from his patient. To few handicaps are we so sensitive as to facial disfigurement, for it at once hinders easy intercourse and reduces our attractive qualities. The public therefore are quickly appreciative of the surgeon's efforts to eradicate or minimise physical stigmata which are so painfully obvious. The author handles his task with zeal and enthusiasm which extend to the smallest details and stamp the book as a scientific work. The basic principles are clearly enunciated. He insists on the need for absence of tension in grafts used to cover defects. For this reason he prefers grafts derived from distant spare areas, such as the front of the arm, rather than flaps drawn across the deficient part from the skin around it. Very sound instruction is given on the need for cell to cell contact of graft and its bed in the Thiersch and Wolfe methods, and this is ensured by an overlying stout compress fixed in place for ten days. The plentiful illustrations are mainly cases of soldiers hideously defaced by the war and remarkably restored, often by monster grafts. The application of methods thus elaborated to civilian cases is given helpfully, as well as the chapter on harelip and cleft palate. The book closes with a lucid description of a bold and apparently effective operation for complete ankylosis of the jaw joint. When so great an authority omits any reference to the treatment of partial ankylosis and derangements of this joint one concludes operation is not generally useful for such conditions.

**Goitre.** By Professor F. de Quervain. Translated from the French by J. Snowman, M.D., M.R.C.P. (with 118 Illustrations and a Bibliographical Appendix). Pp. xii., 247. London: John Bale, Son and Danielsson Ltd. 1924. Price 21s.—Professor de Quervain, of Berne, has in this book provided the reader with a lucid, succinct and practical account of the principles and methods adopted in his clinic for the treatment of goitre. Such an account from such a source will prove deeply interesting to all who have to treat the disease. The surgical anatomy and physiology of the thyroid gland is first considered, then the pathological anatomy and physiology of goitre, then, in order, diagnosis, including skiagraphy, prophylaxis, non-operative, operative and post-operative treatment and prognosis. We note that local is preferred to general anæsthesia and the technique for the production of the former described. The author writes: "The great advantage of this method is that we have the function of the recurrent laryngeal nerve under control at any moment of the operation," and "it does away almost entirely with vomiting." The early ligature of the inferior thyroid arteries in the course of operation, before exposure of the capsule of the goitre, is strongly advised wherever possible. For the removal of goitre tissue a so-called "melon-slice" incision is recommended by which portions of both lobes of the gland and of the isthmus are removed. Hemi-thyroidectomy is reserved for a small minority of cases. A special feature of the book is the number and excellence of the illustrations. The translation reads as if the work had been written primarily in English, and the text is printed in clear type on good paper.

**Postgraduate Lectures.** With Preface by Sir Thos. Clifford Allbutt. Pp. xv., 216. London: John Bale, Sons & Danielsson Ltd. 1922.—The Preface is itself a contribution of much interest and value, and we make no apology for citing the statements of the distinguished writer, who furnishes the most practical critique of the series of lectures in this volume. "The perusal of the lectures in this volume will go far to convince the most self-contained of practitioners that in our time without periodical class teaching and co-operative practice he cannot efficiently equip himself." The lectures of Sir George Savage on "Syphilis and Insanity," of Sir William Hale White on "The Prognosis of Exophthalmic Goitre," of Dr. Wilfred Harris on "Paroxysmal Trigeminal Neuralgia and its Treatment," serve as examples of the selection of subjects dealt with by colleagues who have contributed to our knowledge in such large measure as to constitute them authoritative, but the same may be said with equal justice of each and all the others. The ten lectures were delivered before the Fellowship of Medicine at the Royal Society of Medicine and Sir J. Y. W. MacAlister in his introductory note is justified in stating that "we are indebted to the enterprise of Messrs. Bale" in the production of this the first volume, and which we understand has proved so encouraging that a further volume may shortly be expected.

Pye's **Surgical Handicraft.** Edited and largely re-written by W. H. Clayton-Greene, C.B.E., B.A., M.B., B.C. (Camb.), F.R.C.S. (Eng.). Ninth Edition. Pp. xvi., 619. Illustrations 344, with Plates XIV. Bristol: John Wright & Sons Ltd. 1924. Price 21s.—The new edition of this well-known text-book appears as a weighty volume of over six hundred pages, and it may well be thought that the time has come to see if some reduction in size cannot be managed without impairing the usefulness of the book. It strikes one at once that a section on Poisons is out of place in a surgical manual and, good as it is, it might well go. Moreover, in much of the descriptive writing there is a good deal of unnecessary verbiage, and much space might be saved by courageous use of the blue pencil in reducing this. The book is very freely illustrated, which is all to the good, and the illustrations are on the whole adequate, although some of the blocks which show instruments with bone handles might well disappear from the next edition. The instruction given is sound and, on the whole, complete, and few omissions of importance are to be found. In the Urinary section some reference might be expected to Pezzer's tube, which is in such general use, and something more than a mere reference to tests for renal efficiency would be of value. One more criticism and we have done. Is it not about time that the hot-bath treatment of strangulated hernia finally disappeared from the text-books, for how many students or house surgeons of the present generation have ever seen it carried out, or even heard it mentioned in bedside teaching? Having dealt with certain blemishes, we must state in fairness that they are few and of small importance in comparison with the vast amount of information contained in a book which has been the stand-by of house surgeons and the like for forty years past. We can cordially recommend the present edition as being likely to sustain and increase a reputation which has been steadily built up during that time.

**The Advance of Orthopædic Surgery.** By A. H. Tubby, C.B., C.M.G., M.S. Lond., F.R.C.S. Eng. Pp. xii., 144. London: H. K. Lewis & Co., 1924. Price 7s. 6d. net.—This small volume consists of a reprint of six articles which Mr. Tubby originally wrote for the *Clinical Journal*. As articles they are quite interesting, but they contain little which a student would not find adequately described in any modern work on Orthopædic Surgery, such as the excellent book by Lovett and Jones. In the opinion of the reviewer it would have been better to reserve the articles for inclusion in any future edition of Mr. Tubby's work on *Deformities and Diseases of the Bones and Joints* than to produce them in separate book form.

**Dosage Tables for Deep-Therapy.** By Friedrich Voltz. Pp xii., 98. London: William Heinemann, 1922. Price 10s. 6d.—A useful book for those using the Erlangen methods. It is purely technical and deals with dosage of X-rays. It saves the radiologist a lot of arithmetic by having the tables worked out.

**Ultra Violet Rays in the Treatment and Cure of Disease.** By Percy Hall, M.R.C.S., L.R.C.P. Pp. xvi., 110. London: William Heinemann. 1924. Price 7s. 6d.—This book on the curative value of Ultra Violet Radiations supplies a great need. It sets out clearly and lucidly its uses and abuses, and should be read by all interested in the newer methods of treatment.

